# Deferoxamine Enhanced Mitochondrial Iron Accumulation and Promoted Cell Migration in Triple-Negative MDA-MB-231 Breast Cancer Cells Via a ROS-Dependent Mechanism

**DOI:** 10.3390/ijms20194952

**Published:** 2019-10-08

**Authors:** Chunli Chen, Shicheng Wang, Ping Liu

**Affiliations:** 1 School of Biomedical Engineering, Shanghai Jiao Tong University, Shanghai 200000, China; 2 School of Biomedical Engineering and Med-X Research Institute, Shanghai Jiao Tong University, Shanghai 200000, China

**Keywords:** deferoxamine, breast cancer, iron, mitochondria, ROS

## Abstract

In our previous study, Deferoxamine (DFO) increased the iron concentration by upregulating the expression levels of TfR1 and DMT1 and exacerbated the migration of triple-negative breast cancer cells. However, the mechanisms of iron distribution and utilization in triple-negative breast cancer cells with a DFO-induced iron deficiency are still unclear. In this study, triple-negative MDA-MB-231 and estrogen receptor (ER)-positive MCF-7 breast cancer cells were used to investigate the mechanisms of iron distribution and utilization with a DFO-induced iron deficiency. We found that the mitochondrial iron concentration was elevated in MDA-MB-231 cells, while it was decreased in MCF-7 cells after DFO treatment. The cellular and mitochondrial reactive oxygen species (ROS) levels increased in both breast cancer cell types under DFO-induced iron-deficient conditions. However, the increased ROS levels had different effects on the different breast cancer cell types: Cell viability was inhibited and apoptosis was enhanced in MCF-7 cells, but cell viability was maintained and cell migration was promoted in MDA-MB-231 cells through the ROS/NF-κB and ROS/TGF-β signaling pathways. Collectively, this study suggests that under DFO-induced iron-deficient conditions, the increased mitochondrial iron levels in triple-negative MDA-MB-231 breast cancer cells would generate large amounts of ROS to activate the NF-κB and TGF-β signaling pathways to promote cell migration.

## 1. Introduction

Among cancers affecting females, breast cancer has the highest incidence and a high mortality rate [1,2]. Metastasis is the principal cause of breast cancer-related death [3]. Abnormal iron metabolism may contribute to tumor initiation, growth, and metastasis [4,5,6]. Iron concentrations in tumor tissues and sera from breast cancer patients are much higher than those from healthy people [7,8,9]. The perturbation in the levels of ferritin, which is an intracellular source of iron, is associated with the progression of breast cancer toward a more malignant phenotype [10]. The decreased expression of the cellular iron exporter ferroportin and the increased expression of the iron importers transferrin receptor (TfR) and lipocalin 2 are associated with poor prognosis in breast cancer [11,12,13]. Therefore, iron depletion, through a combination of reduced iron uptake and increased iron export, will become a viable method of cancer therapy.

Iron chelators, originally designed to treat iron overload, can be used to prevent tumor progression in patients [14,15,16]. Deferoxamine (DFO) is the first iron chelator to be used as an anticancer drug [15]. Numerous studies have shown its antiproliferative activity against various tumor types [15,17,18,19]. Conversely, some studies have shown that DFO promotes the invasion and migration of some aggressive cancer cells, such as colorectal cancer cells, glioblastoma cells, and triple-negative breast cancer cells [20,21,22], and these results indicate that DFO has different effects on the different cell phenotypes. We recently identified that DFO reduced the intracellular iron levels in estrogen receptor (ER)α-positive breast cancer cells while enhancing the intracellular iron levels and cell migration in triple-negative breast cancer cells [23]. Further study showed that DFO could increase the expression of iron uptake-related proteins, such as TfR1 and DMT1, in triple-negative breast cancer cells; these proteins were involved in increasing the intracellular iron uptake by activating the IL-6/PI3K/AKT pathways [24]. However, under DFO-induced iron deficiency, the mechanism of iron distribution and utilization in triple-negative breast cancer cells remains unclear.

In the present study, aggressive triple-negative breast cancer cells (MDA-MB-231 cells) and non-aggressive ERα-positive breast cancer cells (MCF-7 cells) were used as cellular models. The effects of DFO treatment on iron distribution and utilization in MDA-MB-231 and MCF-7 cells were explored. Our study showed that DFO reduced mitochondrial iron accumulation in MCF-7 cells and enhanced mitochondrial iron accumulation in MDA-MB-231 cells. The cellular and mitochondrial reactive oxygen species (ROS) levels in MDA-MB-231 and MCF-7 cells increased after DFO treatment. However, DFO-induced mitochondrial and cellular ROS inhibited the viability of MCF-7 cells but promoted the migration of MDA-MB-231 cells through the TGF-β and NF-κB signaling pathways.

## 2. Results

### 2.1. DFO Regulated the Mitochondrial Iron Concentrations in MDA-MB-231 and MCF-7 Cells

**Figure 1:**
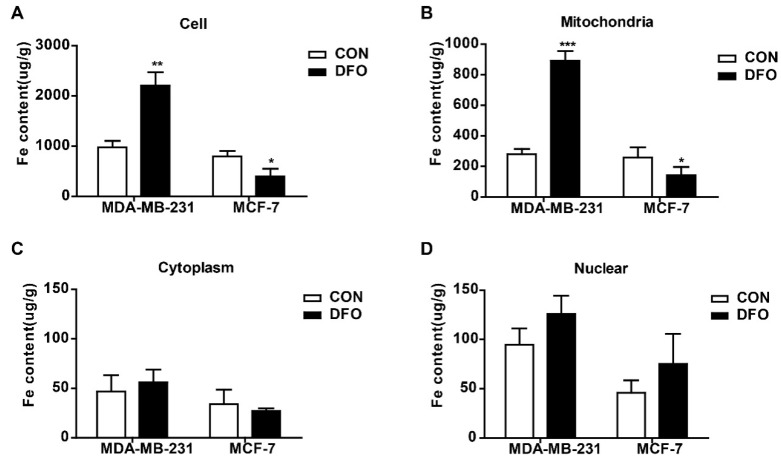
Deferoxamine (DFO) regulated intracellular iron distribution in MDA-MB-231 and MCF-7 cells. MDA-MB-231 and MCF-7 cells were cultured with or without 200 µM DFO for 24h, and (**A**) iron concentrations in MDA-MB-231 cells and MCF-7 cells were measured by ICP-MS. Mitochondria and nuclear were isolated, and iron concentrations of (**B**) mitochondria, (**C**) cytosol, and (**D**) nuclear were measured by ICP-MS. Data were shown as mean ± SD. * versus the control group. *n* = 3, * *p* < 0.05, ** *p* < 0.01, *** *p* < 0.001. ICP-MS: Inductively coupled plasma mass spectroscopy.

Our previous study showed that DFO enhanced the intracellular iron levels in triple-negative breast cancer cells, leading to cell migration [23]. However, the intracellular distribution of the increased iron accumulation in triple-negative breast cancer cells after DFO treatment was unknown. In this study, aggressive triple-negative breast cancer MDA-MB-231 cells and non-aggressive ERα-positive breast cancer MCF-7 cells were used to determine the distribution of iron in breast cancer cells after DFO treatment. The mitochondria and nuclei were isolated, and the iron concentrations in the mitochondria, cytosol, and nucleus were measured by using inductively coupled plasma-mass spectrometry (ICP-MS). Consistent with the results of previous studies [22], after cells were treated with 200 μM DFO for 24 h, the iron concentration in MDA-MB-231 cells was markedly increased, while the iron concentration in MCF-7 cells was significantly decreased (Figure 1A). Interestingly, DFO treatment elevated the mitochondrial iron concentration in MDA-MB-231 cells and lowered the mitochondrial iron concentration in MCF-7 cells (Figure 1B). However, the iron concentrations in the cytosol and nuclei of MDA-MB-231 and MCF-7 cells were not changed after DFO treatment compared with those in the cytosol and nuclei of the control cells (Figure 1C,D). The results indicated that DFO caused the opposite effect in regulating the mitochondrial iron concentrations in non-aggressive ERα-positive MCF-7 cells and aggressive triple-negative MDA-MB-231 breast cancer cells. After DFO treatment, the mitochondrial iron concentration in MCF-7 cells was depleted; conversely, the mitochondrial iron concentration in MDA-MB-231 cells was increased. That is, the increased intracellular iron content in aggressive triple-negative MDA-MB-231 breast cancer cells was mainly distributed in mitochondria after DFO treatment.

### 2.2. The Changes in Mitochondrial Iron Metabolism in MDA-MB-231 and MCF-7 Cells after DFO Treatment

**Figure 2:**
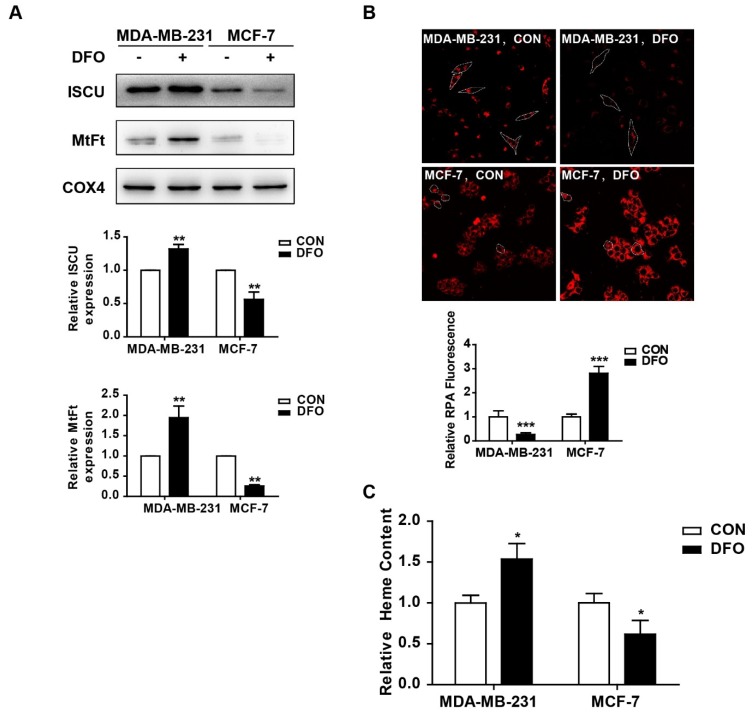
DFO regulated mitochondrial iron metabolism in MDA-MB-231 and MCF-7 cells. MDA-MB-231 and MCF-7 cells were treated with or without 200 μM DFO for 24 h. (**A**) The protein levels of ISCU and MtFt in mitochondrial lysate were detected by western blotting. The results were summarized in the bar graph. (**B**) The level of chelatable mitochondrial iron was measured by RPA. (**C**) The level of heme was measured as described in Materials and Methods. Dashed lines indicate the boundary of one cell. * versus the control group. *n* = 3, * *p* < 0.05, ** *p* < 0.01, *** *p* < 0.001. RPA: Rhodamine B4-((1,10-phenanthroline-5-yl) aminocarbonyl) benzyl ester.

Mitochondria are the major hubs of iron utilization and accumulation [25]. After being imported into mitochondria, iron can be used for Fe-S cluster (ISC) and heme synthesis or can be stored in mitochondrial ferritin (MtFt), and the chelatable iron in mitochondria forms the mitochondrial labile iron pool [26]. Thus, mitochondrial iron metabolism in MDA-MB-231 and MCF-7 cells was studied after DFO treatment. After DFO treatment, the expressions of the Fe-S cluster scaffold protein (ISCU) and MtFt in the mitochondrial lysate were markedly increased in MDA-MB-231 cells, while they were significantly decreased in MCF-7 cells (Figure 2A). The levels of the mitochondrial labile iron pool were measured by using rhodamine B4-[(1,10-phenanthroline-5-yl) aminocarbonyl]benzyl ester (RPA). The fluorescence of RPA in the mitochondria diminished with labile iron accumulation [27]. The addition of DFO resulted in an increase in RPA fluorescence in MCF-7 cells but a reduction in RPA fluorescence in MDA-MB-231 cells, implying the accumulation of chelatable mitochondrial iron in MDA-MB-231 cells (Figure 2B). Similarly, the levels of heme were obviously increased in MDA-MB-231 cells but decreased in MCF-7 cells after DFO treatment (Figure 2C). All of these data demonstrated that in MDA-MB-231 cells, mitochondrial iron metabolism, and accumulation were enhanced, but in MCF-7 cells, mitochondrial iron metabolism and accumulation were impaired after DFO treatment.

### 2.3. DFO Increased Cellular and Mitochondrial ROS in MDA-MB-231 and MCF-7 Cells

**Figure 3:**
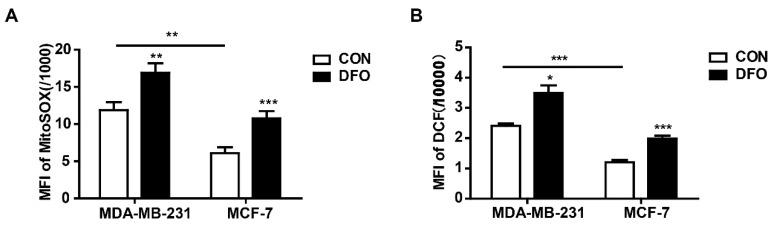
DFO increased intracellular and mitochondrial ROS in MDA-MB-231 and MCF-7 cells. MDA-MB-231 and MCF-7 cells were treated with or without 200 μM DFO for 24 h. (**A**) Mitochondrial ROS levels were assessed by MitoSOX. (**B**) Cellular ROS levels were assessed by DCF-DA. * versus the control group. *n* = 3, * *p* < 0.05, ** *p* < 0.01, *** *p* < 0.001.

Mitochondria are the sites of oxygen consumption and electron transport, and the redox activity of mitochondrial chelatable iron catalyzes Fenton reactions, resulting in the production of ROS [28]. Moreover, as a hypoxia-mimetic agent, DFO induces ROS generation by simulating a hypoxic environment [29,30]. To explore whether DFO induced intracellular and mitochondrial ROS accumulation in MDA-MB-231 and MCF-7 cells, cells were treated with carboxyl-2′,7′-dichlorofluorescein diacetate (DCFH-DA) and MitoSOXTM Red (MitoSOX), respectively. The levels of cellular ROS can be determined by detecting the fluorescence of DCF, and MitoSOX can be used to specifically detect the ROS levels in mitochondria. The results showed that the intracellular and mitochondrial ROS levels were significantly increased in MDA-MB-231 and MCF-7 cells after DFO treatment compared to control cells, but there were higher levels of intracellular and mitochondrial ROS in MDA-MB-231 cells than in MCF-7 cells (Figure 3). We suggested that the increased mitochondrial chelatable iron promoted the production of ROS in DFO-treated MDA-MB-231 cells, but in MCF-7 cells, DFO was a hypoxia-mimetic agent that functioned as a metabolic stressor to increase the ROS levels.

### 2.4. The Effects of DFO on the Cell Viabilities of MDA-MB-231 and MCF-7 Cells

**Figure 4:**
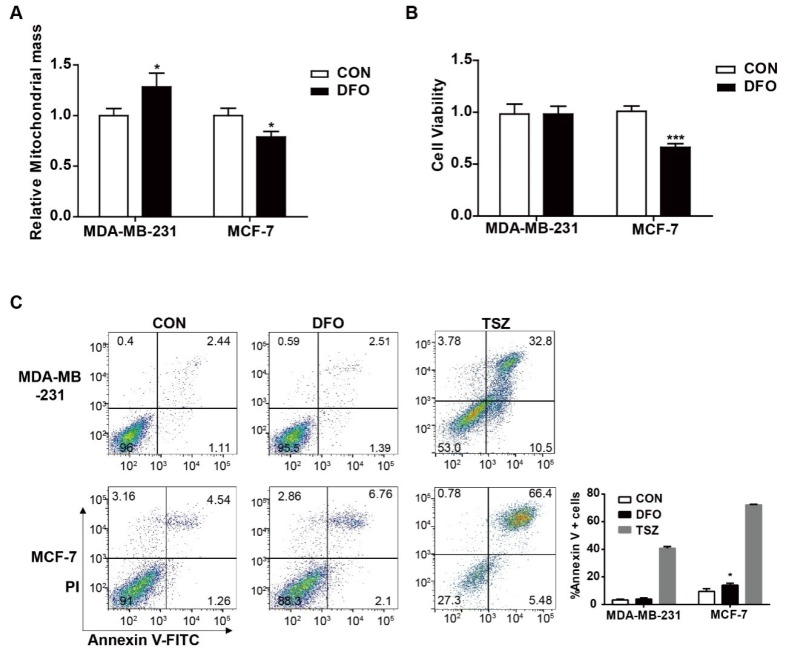
The effect of DFO on mitochondrial biogenesis and cell viability in MDA-MB-231 and MCF-7 cells. MDA-MB-231 and MCF-7 cells were treated with or without 200 μM DFO for 24 h. (**A**) Mitochondrial mass was assessed by MitoTracker Deep Red FM. (**B**) Cell viability was assessed by CCK-8. (**C**) Apoptotic cells were quantified by using Annexin V-FITC. TSZ was used as a positive control. Data were shown as mean ± SD. * versus the control group. *n* = 3, * *p* < 0.05, *** *p* < 0.001.

The above results showed that DFO had different effects on mitochondrial iron concentrations in MDA-MB-231 and MCF-7 cells (Figure 1B). A mitochondrion performs bioenergetic, biosynthetic, and regulatory functions and plays a central role in iron metabolism in cells [25]. We hypothesized that DFO treatment could alter the cell viabilities of MDA-MB-231 and MCF-7 cells. To address this hypothesis, mitochondrial biogenesis, cell viability, and apoptosis were examined after the addition of 200 μM DFO (Figure 4). After DFO treatment, the mitochondrial mass was obviously increased in MDA-MB-231 cells compared to untreated MDA-MB-231 cells. Conversely, mitochondrial mass in DFO-treated MCF-7 cells was decreased compared to that in untreated MCF-7 cells (Figure 4A). After DFO treatment, the cell viability and apoptosis rates of MDA-MB-231 cells were not changed compared to those of untreated cells, while the cell viability of MCF-7 cells was significantly decreased and apoptosis was markedly increased in MCF-7 cells (Figure 4B,C). The supplementation of growth medium with the Necroptosis Inducer Kit with TSZ (TSZ) served as a positive control. The results showed that DFO induced cell death and reduced mitochondrial biogenesis in MCF-7 cells, while DFO treatment induced no significant cytotoxicity and increased mitochondrial biogenesis in MDA-MB-231 cells; these effects contributed to the DFO-induced decrease in iron concentration in MCF-7 cells and the increase in iron concentration in MDA-MB-231 cells.

### 2.5. DFO-Induced Cellular and Mitochondrial ROS Promoted Cell Death in MCF-7 Cells But Had No Effect on Cell Viability in MDA-MB-231 Cells

**Figure 5:**
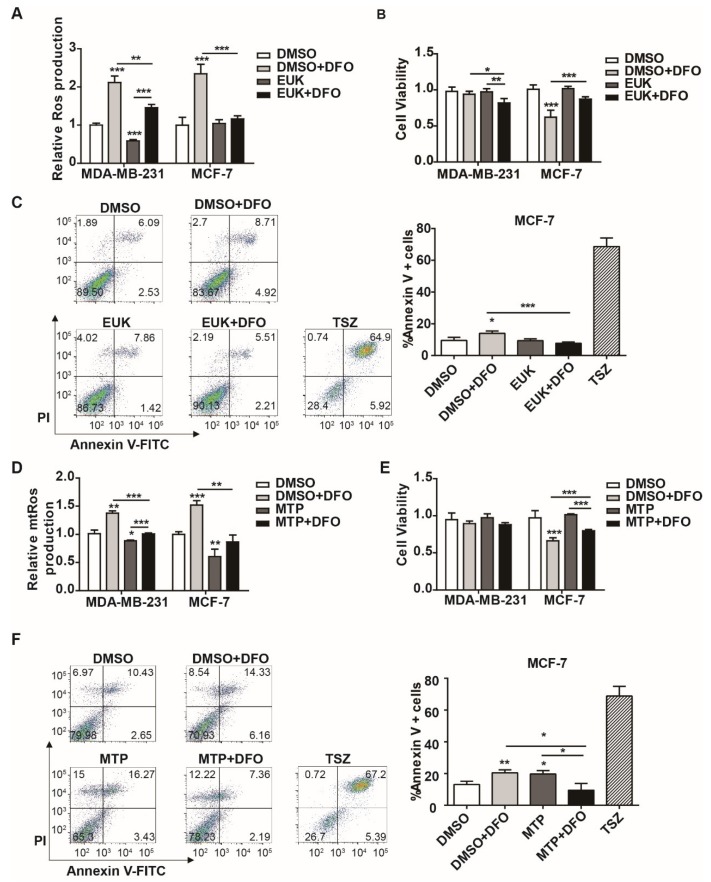
Scavenging reactive oxygen species (ROS) attenuated DFO-induced cytotoxicity in MCF-7 cells. MDA-MB-231 and MCF-7 cells were pretreated with EUK-134 for 1 h, then treated with or without DFO for 24 h. (**A**) Cellular ROS levels were assessed by DCF-DA. (**B**) Cell viability was assessed by CCK-8. (**C**) Apoptotic cells were quantified by using Annexin V-FITC. MDA-MB-231 and MCF-7 cells were pretreated with Mito-TEMPO for 1 h, then treated with or without DFO for 24 h. TSZ was used as a positive control. (**D**) Mitochondrial ROS levels were assessed by MitoSOX. (**E**) Cell viability was assessed by CCK-8. (**F**) Apoptotic cells were quantified by using Annexin V-FITC. TSZ was used as a positive control. * versus the control group. *n* = 3, * *p* < 0.05, ** *p* < 0.01, *** *p* < 0.001. EUK: EUK-134; MTP: Mito-TEMPO; TSZ: Necroptosis Inducer Kit with TSZ.

The above results showed that DFO induced cellular and mitochondrial ROS production in MDA-MB-231 and MCF-7 cells (Figure 3). However, DFO showed a significantly increased cytotoxicity against MCF-7 cells compared to that against MDA-MB-231 cells (Figure 4). We hypothesized that DFO-induced ROS led to cytotoxicity against MCF-7 cells. To prove the hypothesis, cellular ROS was scavenged by the SOD/catalase mimetic EUK-134, and the effects of EUK-134 on cell viability and apoptosis in MDA-MB-231 and MCF-7 cells were evaluated. After 20 μM EUK-134 treatment, the ROS levels in MDA-MB-231 cells were reduced, while the ROS levels in MCF-7 cells were not significantly changed (Figure 5A). After EUK-134 treatment, both DFO-treated MDA-MB-231 and MCF-7 cells displayed lower levels of ROS than the cells in the DFO treatment group. The cell viabilities of MDA-MB-231 and MCF-7 cells were not changed by EUK-134 treatment (Figure 5B). Compared to the cell viability after DFO treatment, the viability of MDA-MB-231 cells decreased after treatment with 20 μM EUK-134 and DFO (Figure 5B). However, EUK-134 treatment reversed the DFO-inhibited cell viability of MCF-7 cells (Figure 5B). Moreover, the DFO-induced apoptosis of the MCF-7 cells was reversed by EUK-134 treatment (Figure 5C). These data indicated that DFO-induced ROS inhibited cell viability and promoted apoptosis in MCF-7 cells; conversely, DFO-induced ROS had no effect on cell viability in MDA-MB-231 cells.

Mitochondria are the major source of ROS [31]. In addition to the mitochondrial respiratory chain, the labile iron in mitochondria is also the primary site of ROS production. To evaluate the role of mitochondrial ROS in DFO-induced cytotoxicity, mitochondrial ROS were scavenged by a mitochondrial-specific antioxidant (Mito-TEMPO), and the effects of Mito-TEMPO on cell viability and apoptosis were examined in MDA-MB-231 and MCF-7 cells. After 100 μM Mito-TEMPO treatment, the mitochondrial ROS levels were reduced in MDA-MB231 cells and MCF-7 cells (Figure 5D). The DFO-induced mitochondrial ROS levels were also reduced in MDA-MB-231 and MCF-7 cells (Figure 5D). The viabilities of MDA-MB-231 and MCF-7 cells were not significantly changed after exposure to 100 μM Mito-TEMPO (Figure 5E). Compared to DFO treatment alone, DFO and Mito-TEMPO treatment increased cell viability and decreased apoptosis in MCF-7 cells, while the viability of MDA-MB-231 cells was not changed significantly (Figure 5E,F). The results suggested that DFO-induced mitochondrial ROS inhibited cell viability and promoted apoptosis in MCF-7 cells, while there was no significant change in the cell viability of MDA-MB-231 cells. These data indicated that DFO-induced cellular and mitochondrial ROS contributed to cell death in MCF-7 cells, while DFO-induced ROS had no effect on cell viability in MDA-MB-231 cells.

### 2.6. DFO-Induced ROS were Essential for Cell Migration in MDA-MB-231 Cells

**Figure 6:**
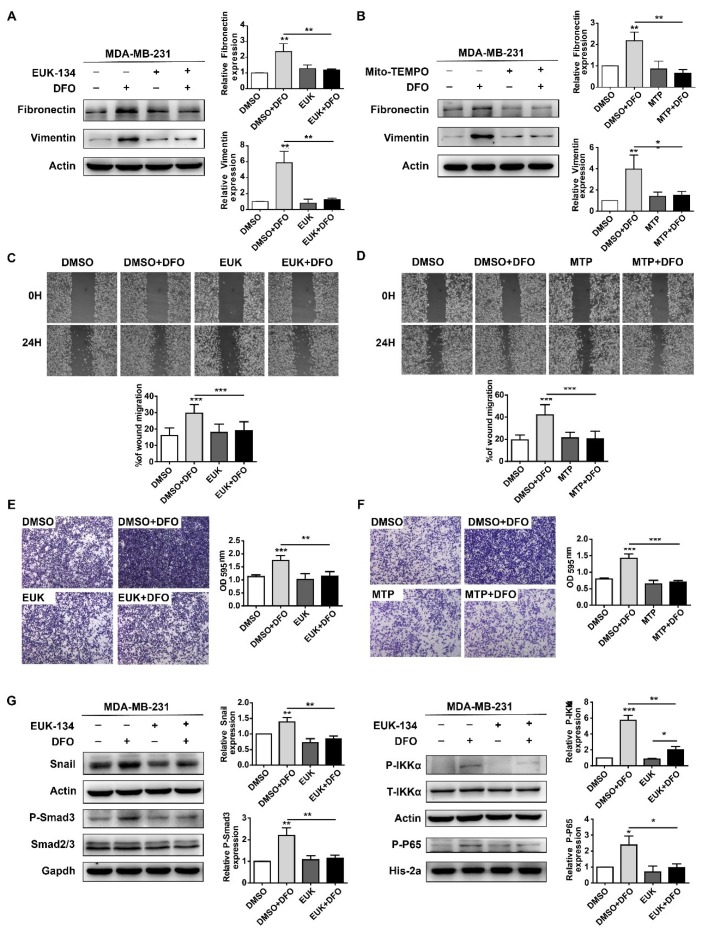
Scavenging ROS reversed DFO-enhanced migration in MDA-MB-231 cells through TGF-β signaling and NF-κB signaling pathways. MDA-MB-231 cells were pretreated with EUK-134 or Mito-TEMPO for 1 h, then co-incubated with or without DFO for 24 h. (**A**) The expressions of fibronectin and vimentin were assessed by western blotting. (**B**) The expressions of fibronectin and vimentin were assessed by western blotting. (**C**,**D**) Cells were allowed to migrate in the wound-healing assays. (**E**,**F**) Cells were allowed to migrate in transwell assays. The images were acquired using an upright microscope with the 5× objective. (**G**) MDA-MB-231 cells were pretreated with EUK-134 for 1 h, then treated with or without DFO for 24 h. The expressions of Snail, phosphate-Smad3, phosphate-IKKα, and phosphate-P65 were assessed by western blotting. Data were shown as mean ± SD. * versus control group. *n* = 3, * *p* < 0.05, ** *p* < 0.01, *** *p* < 0.001. EUK: EUK-134; MTP: Mito-TEMPO.

As a crucial second messenger, ROS regulate a variety of signaling pathways involved in cell migration [32,33]. The above results showed that DFO-induced cellular ROS maintained cell viability in MDA-MB-231 cells (Figure 5). Previously, DFO enhanced the migration of MDA-MB-231 cells by activating both the TGF-β signaling pathway and the TNF-α-dependent NF-κB signaling pathway [23]. To investigate whether DFO-induced ROS affected the migration of MDA-MB-231 cells through the TGF-β signaling pathway and the TNF-α-dependent NF-κB signaling pathway, 20 μM EUK-134 and 100 μM of Mito-TEMPO were used to scavenge cellular and mitochondrial ROS, respectively. Consistent with the results of a previous report [23], after DFO treatment, the expression of fibronectin and vimentin in MDA-MB-231 cells was increased (Figure 6A,B), and the migration of MDA-MB-231 cells was enhanced (Figure 6C–F). EUK-134 and Mito-TEMPO did not change the expression of fibronectin or vimentin in MDA-MB-231 cells, and the migration of MDA-MB-231 cells was also not altered (Figure 6A,B). Compared to DFO treatment alone, pretreatment with EUK-134 or Mito-TEMPO for 1 h and DFO treatment for 24 h decreased the expression of fibronectin and vimentin, and the migration of MDA-MB-231 cells was inhibited (Figure 6A–F). These results suggested that DFO-induced cellular and mitochondrial ROS promoted the migration of MDA-MB-231 cells. After EUK-134 treatment, the expression of Snail, phosphate-Smad3 (p-Smad3), phosphate-IKKα (P-IKKα), and phosphate-P65 (P-P65) were not changed in MDA-MB-231 cells (Figure 6G), which indicated that the TGF-β signaling pathway and the NF-κB signaling pathway were not changed in MDA-MB-231 cells after EUK-134 treatment. Compared with DFO treatment alone, DFO and EUK-134 treatment reduced the protein levels of Snail and P-Smad3 (Figure 6G), and these results indicated that DFO-induced TGF-β signaling pathway activation was inhibited by scavenging ROS. Moreover, consistent with the results of a previous report [23], DFO treatment increased the expression levels of P-IKKα and P-P65 (Figure 6G). However, DFO and EUK-134 treatment decreased the expression levels of P-IKKα and P-P65 (Figure 6G), indicating that ROS wer the upstream signal for the NF-κB signaling pathway. The results suggested that DFO enhanced the migration of MDA-MB-231 cells through the ROS-mediated TGF-β signaling and TNF-α-dependent NF-κB signaling pathways.

## 3. Discussion

In the present study, we identified that (1) the iron chelator DFO reduced mitochondrial iron accumulation and iron metabolism in ERα-positive MCF-7 breast cancer cells and significantly increased mitochondrial iron accumulation and iron metabolism in triple-negative MDA-MB-231 breast cancer cells; (2) DFO induced cellular and mitochondrial ROS production in MDA-MB-231 and MCF-7 cells, but DFO-induced cellular and mitochondrial ROS contributed to apoptosis of MCF-7 cells, while DFO-induced cellular and mitochondrial ROS aggravated the migration of MDA-MB-231 cells through the ROS-mediated TGF-β signaling pathway and the NF-κB signaling pathway.

Our previous study showed that the iron chelator DFO depleted the iron levels in non-aggressive ERα-positive breast cancer cells but resulted in increased iron levels by upregulating the expression of TfR1 and DMT1 in aggressive triple-negative breast cancer cells [23]. Mitochondria are the major sites of intracellular iron distribution. Mitochondrial iron is primarily utilized in iron-sulfur cluster biogenesis, mitochondrial ferritin storage, heme synthesis, and chelatable labile iron [25]. In this study, DFO resulted in increased mitochondrial iron concentrations in MDA-MB-231 cells. After DFO treatment, the heme levels and chelatable mitochondrial iron levels were enhanced in MDA-MB-231 cells. Increase in ISCU and MtFt levels in DFO-treated MDA-MB-231 cells were observed, which would be due to an increase in mitochondrial mass. In addition, iron in mitochondria can be used for Fe-S cluster (ISC) or can be stored in mitochondrial ferritin (MtFt) to prevent iron induced toxicity [26], thus the increased levels of ISCU and MtFt would be induced by the increased mitochondrial iron content in DFO-treated MDA-MB-231 cells. This suggested that DFO-induced mitochondrial iron accumulation might play an essential role in supporting cell growth in triple-negative MDA-MB-231 breast cancer cells.

The increased chelatable iron catalyzes excess Fenton reactions, contributing to the production of ROS [34,35,36,37,38]. Moreover, as a hypoxia-mimic compound, DFO increased ROS accumulation in various cells [20,21,39]. In the present study, DFO induced cellular and mitochondrial ROS generation in MCF-7 and MDA-MB-231 cells. We considered that the increased ROS in triple-negative breast cancer MDA-MB-231 cells was produced by the DFO-induced increased iron content, while the increased ROS in ER-positive MCF-7 cells was induced by a DFO-triggered hypoxic environment, which needed further exploration.

Due to their dualistic nature, ROS can act as “good” and “bad” molecules depending on cell type and the amount of ROS [40,41]. A moderate increase in ROS may promote cell proliferation and survival. However, when the increase in ROS reaches a certain level (the toxic threshold), it may overwhelm the antioxidant capacity of the cell and trigger cell death [42]. We showed that DFO-induced ROS and mitochondrial ROS have different effects on cell viability and apoptosis in MCF-7 cells and MDA-MB-231 cells. Scavenging cellular and mitochondrial ROS suggested that DFO-induced ROS contributed to cell death in MCF-7 cells but maintained cell viability in MDA-MB-231 cells, which suggested that DFO-induced ROS levels were below the tolerability threshold in MDA-MB-231 cells but above that in MCF-7 cells.

In malignant breast cancer cells, alterations in some metastatic genes are associated with elevated ROS generation [43]. ROS are critical signaling intermediaries to control TGF-β signaling and NF-κB signaling [44,45]. In our previous study, DFO-increased iron concentrations promoted cell migration through both the TGF-β signaling pathway and the TNF-α-induced NF-κB signaling pathway in MDA-MB-231 cells [23]. To further test whether DFO-induced TGF-β signaling and NF-κB signaling activation were specifically regulated by ROS, a specific scavenger of ROS was used in MDA-MB-231 cells. The phosphorylation of Smad3, IKKα and P65 was reduced after scavenging DFO-induced ROS accumulation, which suggested that DFO enhanced MDA-MB-231 cell migration through ROS-mediated TGF-β signaling and NF-κB signaling activation.

In this study, we found that iron content in mitochondria was increased after DFO treatment. The increased chelatable iron can induce the generation of ROS through Fenton reactions [34,35,36,37,38], we suggest that under DFO-induced iron-deficient conditions, the increased mitochondrial iron in triple-negative MDA-MB-231 breast cancer cells would induce the increase of mitochondrial ROS to activate the NF-κB and TGF-β signaling pathways, which is required for promoting cell migration. However, the relationship among the increased mitochondrial iron content, the production of ROS, and cell migration need to be studied further.

Our current study also found that DFO increased mitochondrial biogenesis in MDA-MB-231 cells. In contrast, DFO significantly decreased mitochondrial biogenesis in MCF-7 cells. Mitochondria are the most important energy production organelles. High levels of mitochondrial biogenesis are related to enhanced tumor progression and poor overall survival in gastric cancer patients [46]. Increased mitochondrial biogenesis is coupled with enhanced energy production to support cell migration and invasion in cancer cells [47]. Thus, we suggest that the increased mitochondrial mass in DFO-treated MDA-MB-231 cells would supply more energy for cell migration, which requires further study.

However, in the present study, there were some limitations of our experimental set up: Monitoring and measurement of DFO and DFO-iron complex should be needed to support our findings and proposed mechanisms of action of DFO; the quantification and importance of ferric iron in cytoplasm and mitochondria should be taken into consideration; other iron chelators, such as deferiprone (DFP) and deferasirox (DFX), have been clinically approved for iron overload disorders. The mode of action of the other chelating drugs should facilitate the clarification of the role of iron to explore the possibility of using chelation as a possible mode of anticancer therapy [48]. In addition, we did not illustrate how DFO could cause iron deficiency and increase mitochondrial iron. DFO-induced high expression of TfR1 and DMT1 enhanced iron uptake by activating IL-6/PI3K/AKT pathway in MDA-MB-231 breast cancer cells [24], we supposed that the increased iron in the cytoplasm would be stored in ferritin, and under DFO-induced iron-deficient condition, the increased mitochondrial iron in MDA-MB-231 cells would be derived from ferritin by lysosomal degradation.

Furthermore, because in our other study, we demonstrated that ferric ammonium citrate treatment promoted cell migration in MDA-MB-231 cells but not in MCF-7 cells, and the mechanism was also explored (unpublished data), mode of action of iron in the absence of chelators was not identified in this study.

In summary, the present study provided the new conclusion that DFO affects breast cancer cell viability and migration in a ROS-dependent manner. The study identified that DFO-induced ROS enhanced the migration of triple-negative MDA-MB-231 breast cancer cells through the ROS-mediated TGF-β and NF-κB signaling pathways.

## 4. Materials and Methods

### 4.1. Cell Culture

The non-aggressive ERα-positive MCF-7 breast cancer cell line and aggressive triple-negative MDA-MB-231 breast cancer cell line were purchased from the American Type Culture Collection (Manassas, VA, USA). The cells were cultured at 37 °C in an atmosphere of 5% CO_2_ and 95% air in Dulbecco′ s Modified Eagle Medium (DMEM), supplemented with 10% fetal bovine serum.

### 4.2. Mitochondrial and Cytosolic Fractionation Collection

Mitochondria and cytosol were separated using the Cell Mitochondria Isolation Kit (Beyotime Jiangsu, China). Briefly, the collected cells were resuspended and homogenized in extraction buffer at 4 °C for 15 min. The suspensions were centrifuged at 800× *g* for 10 min at 4 °C. The pellets containing the cytosolic fractions were collected. The supernatants were centrifuged at 11,000× *g* for 10 min at 4 °C. The pellets containing the mitochondria fractions were collected.

### 4.3. Nuclear Extraction

The nuclear fraction was isolated using the Nucl-Cyto-Mem Preparation Kit (Applygen Technologies Inc., Beijing China). Collected cells were washed with ice-cold PBS and stirred in a homogenizer with cytosol extraction reagent. Then, the homogenates were centrifuged at 800× *g* for 5 min. The pellets were washed with nuclear extraction reagent twice to obtain the nuclear fraction.

### 4.4. Inductively Coupled Plasma-Mass Spectrometry (ICP-MS) Analysis

Samples were digested in 70% trace metal basis nitric acid (Sigma-Aldrich, St Louis, MO, USA) and then diluted with double distilled H_2_O. The iron contents of the samples were determined by ICP-MS (Thermo Fisher Scientific, Bremen, Germany). The iron concentrations were normalized to the weight of each sample. Data were representative of 3 separate experiments.

### 4.5. Heme Content Determination

For the determination of the cellular heme levels, the cells were collected and lysed in a RIPA buffer. Then, 10 μL of protein was mixed with 200 μL of 2 mol/L oxalic acid, and the solution was heated to 95 °C for 30 min. The samples were then centrifuged for 10 min at 1000× *g* at 4 °C to remove the debris. The supernatant was moved to an opaque 96-well culture plate, and the fluorescence was assessed at 405/600 nm by using a Spectra Max Gemini fluorescence microplate reader. The data were normalized to the protein concentration.

### 4.6. Mitochondrial Fe^2+^ Detection

Detection of free mitochondrial iron was performed using RPA (Squarix Biotechnology GmbH, Marl, Germany). Cell culture media were removed and replaced with RPA (0.5 μM for 20 min in HBSS (Biotime) at 37 °C). Cells were washed twice with HBSS and incubated for an additional 15 min in a dye-free buffer at 37 °C. RPA red fluorescence was determined by quantitative laser scanning confocal microscopy (Leica TCS SP5, Weztlar, Germany).

### 4.7. Flow Cytometry for ROS, Mitochondrial ROS Detection, and Mitochondrial Mass Detection

The intracellular ROS and mitochondrial-generated superoxide contents were measured by DCFH-DA (Biotime) and MitoSOX Red (Molecular Probes-Invitrogen, Rockford, IL, USA). Cells were collected and incubated with 10 μM DCFH-DA in fresh culture medium for 20 min at 37 °C or 3 μM MitoSOX in HBSS for 10 min. The fluorescence intensity of DCF was measured by flow cytometry with the excitation source set at 488 nm and the emission source set at 525 nm, and the MitoSOX fluorescence intensity was measured by flow cytometry with the excitation source set at 510 nm and the emission source set at 580 nm, respectively. Mitochondrial mass was assessed by MitoTracker Deep Red FM (Invitrogen). MitoTracker Deep Red fluorescence intensity was measured by flow cytometry with the excitation source set at 644 nm and the emission source set at 665 nm.

### 4.8. Cell Viability Assay

Cells (1 × 10^4^) were seeded into 96-well culture plates overnight at 37 °C. Then, the cells were pretreated for 1 h with EUK-134 (Cayman Chemical Company, Ann Arbor, MI, USA) or Mito-TEMPO (Enzo Life Sciences, Farmingdale, NY, USA) and then co-incubated with or without 200 µM DFO (Sigma) for 24 h. Following the incubation period, 100 μL DMEM including 10 μL Cell Counting Kit-8 solution (CCK-8 reagent, Dojindo, Kumamoto, Japan) was added to each well; the samples were incubated for 2 h at 37 °C. Cell viability was analyzed by measuring the absorbance at 450 nm with a microplate reader (Synergy 2 Multimode Microplate Reader, Bio Tek, Winooski, VT, USA).

### 4.9. Apoptosis Assays

Apoptotic cells were quantified using the FITC Annexin V Apoptosis Detection Kit (BD Pharmingen, San Diego, CA, USA). Collected cells were resuspended in binding buffer and incubated with Annexin V-FITC and PI for 15 min at room temperature in the dark. The stained cells were analyzed by flow cytometry within 1 h. As a positive control, the cells were incubated with Necroptosis Inducer Kit with TSZ (Beyotime) for 4 h at 37 °C.

### 4.10. Wound Healing Assay

For the wound healing assay, cells were seeded in 6-well plates until they reached confluence. A wound was created by 200 μL pipette tips and washed twice with PBS. Then, the cells were pretreated for 1 h with 20 µM EUK-134 or 100 µM Mito-TEMPO and then co-incubated with 200 µM DFO in DMEM without serum for 24 h. Images were acquired by using an inverted microscope with a 4× objective at 0 h and 24 h.

### 4.11. Migration Assay

Transwell migration assays were performed in a chamber system, and 3 × 10^5^ cells suspended in 100 µL DMEM without serum were added to the upper chamber of a 6.5 mm (0.8 μm pore size) 24-well transwell (Corning, Corning, NY, USA). DMEM with 10% FBS was placed into the bottom wells. After incubation at 37 °C overnight, the cells were pretreated for 1 h with 20 µM EUK-134 or 100 µM Mito-TEMPO and then co-incubated with 200 μM DFO for 24 h. Then, the upper chamber was washed with PBS 3 times, fixed with 4% methanol for 10 min, and stained with crystal violet. Images were acquired using an inverted microscope (Leica DM2500) with a 5× objective. The remaining crystal violet staining of the migrated cells was dissolved in 33% acetic acid. The OD595 nm of the eluted crystal violet was determined. Each experiment was performed in triplicate.

### 4.12. Western Blot Analysis

Twenty micrograms of protein were resolved by 8% or 10% SDS-PAGE and transferred to a polyvinylidene fluoride (PVDF) membrane. Antibodies against mitochondrial ferritin, fibronectin, vimentin, COX 4, and His-2A were obtained from Abcam. An antibody against ISCU was obtained from Gene Tex. Antibodies against β-actin and GAPDH were obtained from Biotime, and antibodies against Smad2/3, p-Smad3, Snail, p-IKKα, IKKα, and p-p65 were obtained from Cell Signaling Technology. Western blot analysis was performed using the primary antibodies and was detected using the appropriate HRP-labeled secondary antibody (KPL, Milford, MA, USA) and enhanced chemiluminescence (Pierce, Rockford, IL, USA). Each Western blot shown is representative of 3 separate experiments.

### 4.13. Statistical Analysis

The results were expressed as the mean ± standard deviation (SD) of at least three independent experiments. Statistical analyses were conducted with Student’s t-test. A *p*-value of <0.05 was regarded as statistically significant.

## Abbreviations

DFO	Deferoxamine
TfR1	Transferrin receptor
ICP-MS	Inductively coupled plasma mass spectroscopy
MtFt	Mitochondrial ferritin
RPA	Rhodamine B4-[(1,10-phenanthroline-5-yl) aminocarbonyl] benzyl ester
DCF-DA	Carboxyl-2′,7′-dichlorofluorescein diacetate
MitoSOX	MitoSOX™ Red
TSZ	Necroptosis Inducer Kit with TSZ
CCK-8	Cell counting kit-8
